# Combining Acceptance and Commitment Therapy With Adventure Therapy to Promote Psychological Wellbeing for Children At-Risk

**DOI:** 10.3389/fpsyg.2018.01565

**Published:** 2018-08-27

**Authors:** Danielle Tracey, Tonia Gray, Son Truong, Kumara Ward

**Affiliations:** Centre for Educational Research, School of Education, Western Sydney University, Penrith, NSW, Australia

**Keywords:** acceptance and commitment therapy, adventure therapy, wellbeing, mental health, at-risk children

## Abstract

With high rates of psychological distress reported amongst children internationally, the development and evaluation of new program initiatives is critical in order to meet the challenge of this burgeoning issue. Both acceptance and commitment therapy and adventure therapy are emerging as popular strategies to elevate psychological wellbeing. This small-scale program evaluation focuses on nine upper primary school-aged children enrolled in a specialist school in Australia for children with challenging behavior and/or emotional needs. Participants completed a newly developed 8-week intervention entitled ‘ACT in the Outdoors’ which combined key principles of both acceptance and commitment therapy and adventure therapy. The program was evaluated via a combination of pre and post participant psychological measures, and post interviews with participants and teachers. The results of this small-scale preliminary evaluation suggest that a portion of the participating children reported improvements in psychological wellbeing and skill development. Improvements appear to be mitigated by attendance and level of psychological wellbeing upon program entry. Based on this premise, the results suggest that more research is warranted to further understand the potential benefit of this innovative interdisciplinary approach.

## Introduction

High prevalence rates of psychological difficulties amongst children present as an enduring international problem. The most recent World Health Organization’s (World Health Organization [WHO], 2005) inquiry into child mental health affirmed that worldwide, 10–20% of children and adolescents experience mental disorders. This remains a significant issue with WHO’s Mental Health Action Plan 2013–2020 (World Health Organization [WHO], 2013) advocating that there should be an international focus on assisting young people to develop a positive sense of identity and the ability to manage thoughts and emotions to enable full active participation in society.

In Australia, the site of the current study, around one in seven children and adolescents aged 4–17 years exhibit a psychological or behavioral disorder with attention deficit hyperactivity disorder, anxiety, depression and conduct disorder the most prevalent (Lawrence et al., 2015). If unresolved, these childhood difficulties negatively impact on children’s development and the attainment of future productive adult lives (World Health Organization [WHO], 2005). Indeed, robust longitudinal studies have confirmed that anxiety and depressive symptoms in childhood and adolescence significantly predict major depression and mood disorders in adulthood (Reinherz et al., 2003; Roza et al., 2003).

The identification and development of effective resolutions for this burgeoning problem warrants attention. The World Health Organization [WHO] (2005) lamented that the construction of both policies and interventions to support child and adolescent mental health lagged behind efforts made for adults. Given the predictive nature of childhood mental health for adult outcomes, work with children and adolescents necessitates greater consideration. Since the World Health Organization [WHO]’s (2005) deposition, two primary bodies of therapeutic work have emerged with children: Acceptance and commitment therapy and outdoor adventure therapy. The current study utilized both of these approaches and thus a review of their definitions, current application and evidence-base will be presented.

### Acceptance and Commitment Therapy

Acceptance and commitment therapy (ACT) has arisen as an increasingly popular therapeutic approach and is considered as the ‘thirdwave’ of cognitive behavioral therapies (Fletcher and Hayes, 2005). Unlike its predecessors, ACT focuses on a person’s thinking and behavior to achieve a valued and meaningful life, and thereby reduce psychological distress, rather than centring on the control and removal of symptoms of psychopathology in itself (Simon and Verboon, 2016). The key aim of ACT is to increase one’s psychological flexibility, which is defined as the ability to be present in the moment, pursue important values and select behavior that is aligned to these values whilst accepting the presence of unpleasant experiences (Hayes and Strosahl, 2004). ACT identifies six core processes that work together to achieve psychological flexibility: acceptance, defusion, contact with the present moment also known as mindfulness, self-as-context, valuing, and committed action (Hayes et al., 1999). With the significant overlap and intersection among the six core processes, one’s mastery of these processes is typically measured by experiential avoidance as a proxy for psychological inflexibility (Murrell et al., 2015). A review of the empirical evidence underpinning ACT concludes that ACT has shown to be effective at improving a range of problems where experiential avoidance is present. Effect sizes are large both immediately following intervention and at follow-up (Ruiz, 2010).

Although the construction of ACT intervention programs for children is gaining momentum, comparatively little ACT efficacy research has been conducted with children, as opposed to adults and adolescents (Barney et al., 2017). Simon and Verboon (2016) contend that key characteristics of ACT position it as an ideal intervention for children. Namely, the cognitive components of ACT are easier to master than that of cognitive behavioral therapy, metaphors are used as a central strategy instead of literal instructions, the focus of ACT suits preventative work which is typically aimed at children, and children appear to be more receptive to strategies such as mindfulness and acceptance than adults (Goodman and Greenland, 2009).

Nonetheless, the evidence base affirming the effectiveness of ACT for children remains scarce (Simon and Verboon, 2016; Enoch and Dixon, 2017) and given the resource-intensive delivery of ACT and the focus on samples with specific characteristics (i.e., with identified psychological needs), studies tend to rely on single-case or small sample, uncontrolled studies (Coyne et al., 2011). Although the inherent nature of these designs reduces the capacity to make inferences about efficacy with children, these small case studies and program evaluations help to build a broader body of knowledge.

For example, following nine 50-min ACT sessions, three children previously diagnosed with obsessive compulsive disorder (10–11 years) evidenced clinically significant reductions in obsessive compulsive symptoms (Barney et al., 2017). Similarly, Murrell et al. (2015) reported that out of nine children (11–15 years) with attention deficit hyperactivity disorder, learning problems and behavioral problems, approximately one-third demonstrated clinically significant changes in behavioral symptoms. Furthermore, Ghomian and Shairi (2014) employed a quasi-experimental design where 10 children (7–12 years) with chronic pain received an ACT intervention. Results revealed that, compared to the control group, participating children reported increased functioning. Finally, the sustained attention of children (6–12 years) who received six sessions of ACT intervention focusing on present moment activities was improved compared to those who did not receive the intervention (Enoch and Dixon, 2017). Less is known though about the impact on psychological wellbeing such as anxiety and depressive symptoms for children.

### Adventure Therapy

Together with the rise of ACT, adventure therapy (AT) is gaining momentum as a method to remedy psychosocial difficulties through one’s engagement with outdoor activities and experiential learning exercises (Bowen and Neill, 2013). Often used synonymously with a variety of terms, AT is typically underpinned by the following principles: learning through experience; interaction with nature; heightened arousal from perceived risk; focus on positive change for participants; provision of care and support; and group based delivery where group processes themselves form part of the intervention (Gass et al., 2012).

AT presents as a promising approach for developing the psychological wellbeing of children for both its ability to engage children and also documented outcomes in itself. Children demonstrate a preference for outdoor settings, especially those based in nature (Evans, 2006) and AT can offer a more appealing approach to therapeutic intervention (Bowen et al., 2016). That is, children may be reluctant to engage in traditional intervention approaches (Rickwood et al., 2007) that require sitting still, talking, or writing, which dominate therapeutic approaches with adults.

A mounting body of evidence suggests that AT, and associated approaches, can foster short and long-term therapeutic change (e.g., Dickson et al., 2008; Scrutton, 2015; Bowen et al., 2016), although qualitative evaluations tend to deliver more consistent positive results than quantitative evaluations (Scrutton, 2015). There is a need, however, to conduct further evaluations to produce a robust evidence-base of efficacy, especially for its efficacy with children (Bowen et al., 2016). Research examining the impact of AT for children tends to be based on children without clinical diagnoses, so while the designs and sample sizes permit greater statistical investigation of efficacy than what has been achieved for ACT, the results do not always apply directly to children diagnosed with psychological difficulties. For example, Scrutton (2015) surveyed 360 children (10–12 years) who participated in a residential week of outdoor adventure and compared their pre and post personal and social development with a control group of 115. A small positive effect was witnessed after the intervention, but was not maintained at follow-up. Children who reported poorer personal and social skills initially appeared to gain more from the outdoor adventure experiences.

A randomized controlled trial with 120 senior primary age children demonstrated the efficacy of AT for promoting psychological wellbeing (Li et al., 2013). Children received five 75-min sessions plus one full day of adventure-based training and, in comparison to the control group, reported a reduction in depressive symptoms, lower anxiety levels and higher self-esteem. Furthermore, a recent meta-analysis of AT outcomes (Bowen and Neill, 2013) established an effect size of 0.5 with strongest effects for clinical and self-concept measures. Importantly, age moderated the effectiveness of AT programs with stronger outcomes demonstrated for adult participants. Effect sizes are yet to be determined for children.

In sum, a review of the literature signals the potential efficacy of ACT and AT to promote the psychological wellbeing of children. The current study developed and implemented a new program which comprised principles from both ACT and AT. Although the small scale of the study precludes the evaluation of the additive contribution of combining ACT and AT beyond their individual contributions to psychological wellbeing, it provides a description of the new program and preliminary insight into participant and stakeholder perceptions of impact.

### A New Interdisciplinary Approach to Enhance Psychological Wellbeing of Children: *ACT in the Outdoors*

Premised on ACT and AT, the authors constructed a new intervention program entitled *ACT in the Outdoors.* The authors hold complementary expertise including: a registered psychologist with specific ACT training and experience, a specialist in outdoor learning and experiential education approaches, a certified therapeutic recreation specialist with experience in AT, and a certified nature pedagogue specializing in arts-based pedagogies in the outdoors.

Together they delivered each session in outdoor settings, including the beach, park and on nature walks. The children’s school teachers also participated in the sessions. The program was conducted over 8 weekly sessions (with the first seven sessions of 1 h duration and the final session of 2 h duration). **Table 1** presents the sequence of concepts and activities (for a full description of the program see Truong et al., 2018).

**Table 1:** Sequence of concepts and activities in *ACT in the Outdoors.*

Session	Activities	Focus areas
1	Circle. Establishment of parameters for interaction; reiteration of school approaches of Safe, Respect, Learner. Energy rope. Construction of balancing structures with natural materials. Breathing practices. Overview of all sessions and anticipated outcomes.	Introduction and practice of mindfulness and contact with the present moment. Contact with place.
2	Circle and recap on week 1. Checking in with our bodies in space: stretching, breathing and body awareness. Helium stick. Nature walk to identify anger. Exercises for contextualizing negative thoughts drawn on paper and sand.	Willingness to make space for difficult thoughts. Emotions and behaviors associated with adversity and hardship. Changing the way we respond to difficulties. Recognizing the ephemeral nature of negative thoughts.
3	Circle with focus on positive reflections. Checking in with our bodies and emotions. Oz Tag game with rule changes for reflection. Writing then destroying words depicting negative emotions in the sand. Rock skimming (negative thoughts written on rocks).	Being in the moment. Noticing emotions and how they impact on everyday interactions. Breathing to reduce stress and anxiety. Learning to release oneself from difficult thoughts.
4	Circle: reflection on previous weeks with examples of activities enjoyed or positive thoughts. Balloon (with negative thoughts inscribed) sling in groups of 3. Tug of war game. Chinese finger traps. Mindfulness activity with a focus on the body and thoughts.	Objectifying thoughts – as separate from the self – my thoughts are not me. Communication, attempting to reach consensus, group action, disassociation from negative thoughts. Identifying how we hook ourselves on negative thoughts. Awareness of responses to no-win situations: fight or flight – or witnessing and letting go.
5	Circle and energy rope with recap on being safe, respectful and learner. Breath exercises. Values cards exercise. Clay sculptures and storytelling. Human yurt circle.	Awareness of difficult thoughts, and of energy used in discordant emotions. Living a valued life: identifying personal values and goals. Identifying steps to achieve goals. Working together with group support to stay safe and achieve outcomes.
6	Circle and energy rope; reflections on values. Rob the nest game. Beeswax sculptures, storytelling and totems. Values cards exercise. Breathing and mindfulness.	Working with values. Identifying what it means to live a valued life and what detracts from the valued life. Perspectives on problems. Symbols and totems we can use for reminding us of our values. Developing resilience.
7	Circle and energy rope; reflection on totems and symbols. Problem solving with knots, and charcoal drawings. Developing a plan for implementing problem solving actions. Minefield game. Breathing and stretching.	Taking action: practicing techniques to live with emotions and take action toward a valued life. Unhooking from difficult thoughts. Developing trust. Identifying scale of problems, strategies for solving them, weighing of strategies and choosing one for action plan.
8	Nature hike – with experiential activities. Circle –safety review and identification of purpose of walk. B.O.L.D: Breath, Observe, Listen, Decide. Choice point model exercise – fork in the path. Values clarification: paper plane at end of walk. Letter to self regarding following school year. Cutting of energy rope and making slipknot bracelets for all participants.	Focus on values-guided action: commitment strategies for living a valued life, dealing with setbacks and recognizing progress. Willingness to try something new. Recognizing body and emotions working together and the influence awareness can have on both. Identifying strengths, making decisions and self-compassion. Symbolically committing to actions for a valued life.

### The Present Study

The present study aimed to provide a preliminary evaluation of the *ACT in the Outdoors* program on the psychological wellbeing of children who presented with challenging behavioral and/or emotional needs. This objective was addressed through interviews with participating children and their school teachers to gather their perspective on impacts, as well as participating children’s completion of pre and post quantitative measures.

## Materials and Methods

### Design

The present study implemented a multi-method design (Creswell, 2018) combining the administration of both post-intervention qualitative interviews with participating children and their school teachers, and quantitative pre and post-intervention questionnaires with participating children. Together, these methods provide insight into the perspectives of children and teachers about the impact of *ACT in the Outdoors* on the psychological wellbeing of participants.

### Participants

A total of nine children from the *ACT in the Outdoors* program participated in the quantitative component of the study and completed pre and post questionnaires. Participants included eight males and one female with an average age of 11 years 9 months (ranging from 11 years 2 months to 12 years 10 months; five were in Year 5; four were in Year 6). As a result of exhibited behavioral and emotional difficulties, the children were placed in a “School for Specific Purpose” particularly designed to support children with behavioral and/or emotional challenges by offering small class sizes and alternative pedagogies among other strategies. The participants attended this school from 3 to 5 days out of the 5-day school week (with remaining days being completed at their regular local school). Six children identified themselves as Australian; one as New Zealand origin; and two as Aboriginal. Only one student indicated that they spoke a language other than English, namely Spanish. Seven of these children (six males and one female) also participated in a post-intervention interview.

Three teachers from the school (two males and one female) participated in a post-intervention interview. These teachers had detailed knowledge of the children and *ACT in the Outdoors* as they worked with the children at the School for Specific Purpose and accompanied the children during the *ACT in the Outdoors* program.

### Materials and Procedure

Approval for the research was received from the University Human Research Ethics Committee and the New South Wales Department of Education. Full parental and personal consent was attained for the participating children, whilst teachers provided full personal consent. Participation in the research was voluntary and when participation was declined, children were still able to fully participate in the *ACT in the Outdoors* program.

#### Semi-Structured Interviews With Children

Post-intervention interviews were conducted with participating children to gain insight into their perceived changes in psychological wellbeing or skills from before the program to after the program. Questions included: (1) What is your opinion about the weekly sessions? (2) What did you like the most? (3) Which activities did you enjoy, or get the most out of? (4) What could have been improved? (5) Think about what you were like before you started and what you are like now. Have there been any changes? Please explain (6) What are the main messages or lessons you have taken away from our sessions? (7) How do you apply these principles/lessons at home or at school?

#### Semi-Structured Interviews With Teachers

The post-intervention interviews with teachers sought to examine similar topics but from the perspectives of the teachers who had close knowledge of the children within their everyday school environment and of their *ACT in the Outdoors* journey. Teachers were asked to comment on any changes they had observed in the children following their participation in the program.

#### Pre and Post Questionnaires

A battery of pre-existing measures was selected by the researchers to measure children’ psychological wellbeing (i.e., anxiety, depression, impairment caused by anxiety, general school self-concept) and skills (i.e., psychological flexibility, mindfulness).

##### Kessler10

This is a 10-item measure of anxiety and depression symptoms experienced in the past 4 weeks (Kessler et al., 2002). The measure has been used in studies with Australian adolescents and adults. Scores range from 10 to 50 and are classified into four bands: Low: 10–15, Moderate: 16–21, High: 22–29, and Very High: 30–50. The Kessler10 has excellent internal consistency (α = 0.93) (Kessler et al., 2002). Lower scores are more desirable.

##### Children’s anxiety life interference scale

This is a 9-item measure used to assess the level of life interference and impairment associated with anxiety (Lyneham et al., 2013) where lower scores are more desirable.

##### Self-description questionnaire I

This is a measure of multidimensional self-concept with strong reliability and validity (Marsh, 1990). For the current study, the general school factor was administered; it includes ten items about the student’s competence and enjoyment of school in general, on a 5 point Likert-type response scale. Higher scores are more desirable.

##### Avoidance and fusion questionnaire for youth

This is a 17-item child-report measure used to assess psychological inflexibility provoked by cognitive fusion, experiential avoidance, and behavioral ineffectiveness in the presence of negatively evaluated private events (e.g., thoughts, feelings, physical-bodily sensations) (Greco et al., 2008). Lower scores are more desirable.

##### Child acceptance and mindfulness measure

A 10-item measure of mindfulness which assesses the degree to which children and adolescents observe internal experiences, act with awareness, and accept internal experiences without judging them (Greco et al., 2011). Higher scores are more desirable.

### Data Analysis

Interviews were recorded and transcribed verbatim. Guided by the constant comparative method and thematic coding (Creswell, 2012), interview transcripts were reviewed and categories developed based on presented ideas that related to perceived impact. These categories served as the basis to identify common themes across both child and teacher qualitative data sources.

Given the small sample size in the present study, group analyses were not possible. Reliable Change Index (RCI) scores were calculated for each individual’s scores on the five quantitative measures to determine pre and post changes at the individual level (see Zahra et al., 2016 for a review). Jacobson and Truax’s (1991) standard formula was applied whereby scores greater than 1.96 (95% confidence level) were considered significant.

## Results

### Interviews

An analysis of the semi-structured interviews with both children and teachers identified that participation in *ACT in the Outdoors* evidenced the following changes for participating children: self-calming through mindfulness, committing to action, enhanced teamwork and ability to trust others, and showing support and respect for others.

#### Self-Calming Through Mindfulness

The most prominent impact observed by the teachers was the children’s ability to calm themselves when feeling anxious, angry, or frustrated. This was discussed by the teachers generally in relation to mindfulness, and more specifically, the breathing exercises introduced and practiced throughout each session of the program. The following quote demonstrates this finding:

I have noticed a change in [one student] and to some degree [another]...took on the strategies and everything and have used them. I’ve seen [him] use them …he does use the calming…He just blanks out basically. He blanks out and kind of sits there and does his own thing. He just stares at something and I’ve asked him about it and he said ‘I’m meditating,’ which I don’t know if he is or if he’s just blocked out, but it really seems to calm him down when he does it.

Broadly, mindfulness was identified as a new skill acquired by the majority of the participating children. While the children did not specifically use the term ‘mindfulness’, there were numerous references to ‘*taking time out,’* ‘*calming down,’* ‘*breathing*,’ ‘*meditation*,’ and ‘*yoga*,’ within their recollections of what they learned and their perceived behavioral changes as a result of participating in the program. The following interview exchange with Ned^1^ reveals not only what he learned in relation to applying mindfulness strategies, but also provides an example of how he put this new skill into action in his daily school life.

**Researcher:** What do you think you learned?

**Ned:** Control my anger because now I’m like I can control my anger.

**Researcher:** Do you feel like you can recognize when you start to get angry?

**Ned:** Yeah.

**Researcher:** And what do you do now?

**Ned:** Just calm myself now.

**Researcher:** Okay. How do you calm yourself down?

**Ned:** Breathing. Do that breath stuff.

**Researcher:** Can you think of a time that you’ve done that in the past, since we started our sessions?

**Ned:** Tuesday.

**Researcher:** So you started; what happened?

**Ned:** This [student] was just annoying me so much and then I got to a point and I swore. I didn’t even know I swore. I yelled out and I was like so messed, I didn’t even know…But to calm myself down I walked outside and I went to have a drink and I took some breaths.

Similarly, when asked about what he learned through his participation in the program, Ryan responded:

Well it made me behave more better and calmed me down. Like it would teach me how to calm down instead of being all angry and it’s better…

It just made to calm you down, change your actions, make you more better, calm you down, gives you time out, like yoga. Yeah. Makes you behave. Doesn’t make you angry. Doesn’t make you have thoughts of bad stuff.

While breathing and calming exercises are facets within mindfulness and broader ACT principles, the children’s responses indicate that they were particularly beneficial for managing difficult emotions and subsequent negative behavior. Although some children found it challenging or at times boring to participate in these activities, they also identified the value of developing these new skills, with one student commenting that he grew to respect the program over time.

#### Committing to Action

Core principles of ACT, embodied in *ACT in the Outdoors*, are the identification of values and a commitment to actions that help achieve a life guided by these values. During the interviews, the children identified the positive behaviors they need to commit to in order to achieve their valued life. For example, Nathan stated: “*I do more work. I reckon I listen more…I’m way calmer*.” Identified actions mostly involved applying themselves and tempering the aggressive behavior that has been problematic for them throughout their schooling. For example, Ned stated he would like to “*be good and listen to the teachers and just try my hardest.*” Nick emphasized the importance of taking his time with school tasks that he finds difficult, and Ryan said that he would advise himself to “*be calm, do my work*” and listen to the teacher.

The children also suggested making an effort to socialize and get along with their peers as a way to improve their school experience. Half of the participants insisted that they would need to curtail their aggressive behavior such as fighting, smashing windows and lashing out at others. Ryan in particular discussed how his aggressive behavior led him to leave his regular school and that he would have to “*calm down*” and “*make a change*” if he wanted his school experience to improve. Ned indicated that he wanted to “*Make some friends. Don’t be left out. I don’t want to be left out of nothing. Don’t embarrass myself…I just want to start a whole new life*.”

#### Enhanced Teamwork and Ability to Trust Others

Characteristic of AT, many of the program activities required communication and cooperation amongst group members. The teachers commented that such tasks are often challenging for these children and participation in these activities varied. Nonetheless, the development of teamwork and trust in group members was identified by the teachers as a key outcome of *ACT in the Outdoors.* One teacher commented:

I think that what I’ve noticed is that – and we’ve worked on it all year, but it’s becoming more and more – that they work as a team, a lot more teamwork and a lot more trust between them.

Similarly, another teacher reflected on the development of children’s positive rapport and changing relationships with their school teachers as a result of the program:

They’re seeing us as not so much their bosses and being in charge of them. They’re kind of seeing that we’re there to help them and the positive push that this program’s got has really changed their thoughts toward us and it gives us a lot more base knowledge.

#### Showing Support and Respect for Others

The importance of trust is interrelated with the ability to show support and respect for others. This emerged as another theme, and in particular, the storytelling activity, which involved clay sculpting, was a significant antecedent event. When asked to identify an activity that had the most impact on students, one teacher shared:

For me personally it was the clay building. That day was amazing. To see some of the [students] open up and be so honest and there was no competition between stories. It wasn’t ‘Well I’ve done this,’ ‘I’ve done that,’ and ‘I’ve got a worse life than you.’ It was just open and honest what they were saying. So yeah, that was a real highlight of the [program] for me was a couple of the [students] talking so openly and honestly…No one took it as a ‘Look how bad my life is.’ It was more of a ‘This is just what’s happened to me and I trust you guys enough to tell you.’ So the trust in that exercise was amazing.

The level of trust and rapport within the group was viewed positively. The impact of adults putting their trust in the students, as was the case during a trust and obstacle course activity was also viewed as impactful:

I really loved the minefield activity and I think that the kids really love it when we put our trust in them and it really stood out…when the kids were in charge of our safety and they didn’t abuse it. They took it really seriously.

### Pre and Post Questionnaires

At least one RCI score was significant for five of the nine participants (see **Tables 2–6**). Two children exhibited significantly improved general school self-concept; two children reported significantly reduced anxiety and depression; one child reported significantly reduced life interference associated with anxiety; one child evidenced significantly improved mindfulness whilst another exhibited significantly reduced psychological inflexibility.

**Table 2:** Significant reliable change indices, and pre and post scores, for participant 2.

	Pre	Post	RCI
Mindfulness	25	32	−2.56^∗^

^∗^indicates a significant RCI.

**Table 3:** Significant reliable change indices, and pre and post scores, for participant 4.

	Pre	Post	RCI
Anxiety and depression	40	20	−3.30^∗^
General school self-concept	10	23	1.98^∗^

^∗^indicates a significant RCI.

**Table 4:** Significant reliable change indices, and pre and post scores, for participant 5.

	Pre	Post	RCI
Life interference associated with anxiety	25	6	−3.58^∗^
Psychological inflexibility	30	8	−2.04^∗^

^∗^indicates a significant RCI.

**Table 5:** Significant reliable change indices, and pre and post scores, for participant 6.

	Pre	Post	RCI
Anxiety and depression	30	22	−1.98^∗^

^∗^indicates a significant RCI.

**Table 6:** Significant reliable change indices, and pre and post scores, for participant 8.

	Pre	Post	RCI
General school self-concept	27	40	1.98^∗^

^∗^indicates a significant RCI.

## Discussion

This study sought to provide a preliminary evaluation of a new interdisciplinary program entitled *ACT in the Outdoors*, based on a combination of ACT and AT principles, on the psychological wellbeing of primary age children with challenging behavioral and/or emotional needs. The qualitative interviews provide insight about potential impact, as perceived by participating children and their school teachers. Rather than identify changes to psychological wellbeing, both children and teachers recognized that participating children had acquired new skills and new behaviors that may indeed serve as precursors to future improved psychological wellbeing. Changes include: self-calming through mindfulness, committing to action, enhanced team work and ability to trust others, and showing respect for others. The program was buttressed by the inclusion of the school teachers alongside the children and facilitators. Warm and trusting relationships were cultivated between the children and their school teachers which may lead to greater school engagement and a continuation of ACT and AT principles within the school environment.

The pre and post quantitative findings show that five of the nine participating children exhibited a significant improvement in at least one aspect of psychological wellbeing from pre to post intervention. Improvements were in the area of: general school self-concept (two children); anxiety and depression (two children); life interference associated with anxiety (one child); mindfulness (one child); and psychological inflexibility (one child). A closer examination of these significant changes presents some interesting findings that may guide future research and practice. Firstly, it is notable that the five children who exhibited significant changes had attended seven to eight out of eight sessions, whilst those who did not exhibit significant changes only attended five to six out of eight sessions. It appears that significant change may only occur following completion of seven to eight sessions within *ACT in the Outdoors*. Secondly, significant change was witnessed for children who, at pre-test, presented with the highest anxiety and depression; highest life interference associated with anxiety and lowest general school self-concept compared to the remaining children who did not show any significant improvements. It could be hypothesized that *ACT in the Outdoors* may be most effective for children presenting with particularly low levels of psychological wellbeing. These findings mirror previous research which scrutinizes significant change at an individual level rather than group mean differences. For example, Scrutton (2015) found that children who reported poorer personal and social skills initially appeared to gain more from an AT intervention, whilst Murrell et al. (2015) observed that only four out of nine students demonstrated some significant change as a result of an ACT intervention and cited possible feasibility obstacles such as reduced attendance at sessions as undermining impact.

The study design does not permit evaluation of how the combination of ACT and AT may enhance children’s psychological wellbeing above and beyond the delivery of ACT or AT alone. It does, however, provide a preliminary evaluation of *ACT in the Outdoors*. Given the novel interdisciplinary approach adopted within this study to address children’s wellbeing, the authors make the following observations about combining ACT and AT within nature-based experiences for children with challenging behavioral and/or emotional needs. Firstly, the natural environment served as a key facilitator for the mindfulness activities, teaching breathing exercises and connecting to the present. Similarly, the various movement activities where participants interacted with each other and nature supported the introduction of metaphors and the reinforcement of ACT principles. Children’s engagement in the program was generally assisted by both the outdoor environment and the nature and arts-based activities. Nonetheless, some challenges were experienced which are not uncommon for AT, however, possibly more problematized for children presenting with challenging behavioral and/or emotional needs. At times, the outdoor environment presented distractions (e.g., children wanting to move into the ocean whilst at the beach) and the need to manage physical and emotional safety (e.g., children exhibiting a fear of snakes and being in the bush during the nature walk). Facilitators need to be skilled and prepared to not only deliver ACT and AT but responsive to children’s behavior to optimize outcomes for these children in need.

### Limitations

This study offers practitioners an innovative new program which seeks to combine ACT and AT, however, the results derived from the evaluation should be interpreted with caution due to limitations in design. Firstly, no comparison of results for a control group or long-term follow-up measure points were possible with the available funding. Therefore, changes experienced by the children cannot be directly related to their involvement in the program. Secondly, the study relied on a small sample size which prevented the testing of significant changes for participants as a group. Furthermore, this study employed self-report measures (with no input from parents or teachers in the children’s regular local school), which are susceptible to response bias, rather than objective measures of change in psychological wellbeing. As a result, findings should be considered preliminary. Future research should adopt randomized controlled trial methodologies with larger sample sizes to strengthen the evidence of the efficacy of programs such as *ACT in the Outdoors* which adopt an interdisciplinary approach to boosting children’s psychological wellbeing. More importantly, a rigorous experimental design is required to determine the differential impacts of ACT, AT, and ACT combined with AT to advance practice.

## Conclusion

The current study presents a new program entitled *ACT in the Outdoors* based on the interdisciplinary combination of ACT and AT and delivered this program to children with challenging behavioral and/or emotional needs. The evaluation results, although founded on a small scale inquiry, provide encouraging insight into the possible positive impacts on psychological wellbeing and skill development. More importantly, the results may serve as a catalyst for future research in this emerging area of practice. Given the rise of ACT and AT interventions, randomized controlled studies with larger sample sizes are required to establish evidence of their impact on children’s psychological wellbeing. In practice, the present study suggests that there may be a minimum participation level required to achieve significant change, and that this interdisciplinary intervention may be best targeted to children with particularly low levels of psychological wellbeing. Finally, although intervention programs should be conducted by trained facilitators, the researchers encourage the inclusion of children’s school teachers to bolster the impact of the intervention for children in their school environment. Such a strategy may extend the impact of the intervention into their daily learning environment as witnessed in this study.

## Author Contributions

All authors contributed to the study design, *ACT in the Outdoors* implementation and data collection. DT performed the statistical analysis and drafted the manuscript. ST, TG, and KW conducted the qualitative analysis. All authors critically revised the draft manuscript for publication.

## Conflict of Interest Statement

The authors declare that the research was conducted in the absence of any commercial or financial relationships that could be construed as a potential conflict of interest.

## Footnotes

1 Pseudonyms used

## References

[B1] Barney J. Y. Field C. E. Morrison K. L. Twohig M. P. (2017). Treatment of paediatric obsessive compulsive disorder utilizing parent-facilitated acceptance and commitment therapy. *Psychol. Sch.* 54 88–100. 10.1002/pits.21984

[B2] Bowen D. Neill J. Williams I. Mak A. Allen N. Olson C. (2016). A Profile of outdoor adventure interventions for young people in Australia. *J. Outd. Recuit.* 8 26–40. 10.18666/JOREL-2016-V8-I1-7281

[B3] Bowen D. Neill J. T. (2013). A meta-analysis of adventure therapy outcomes and moderators. *Open Psychol. J.* 6 28–53. 10.2174/1874350120130802001

[B4] Coyne L. McHugh L. Martinez E. (2011). Acceptance and commitment therapy (ACT): advances and applications with children, adolescents, and families. *Child Adolesc. Psychiatr. Clin. N. Am.* 20 379–399. 10.1016/j.chc.2011.01.010 21440862

[B5] Creswell J. (2012). *Qualitative Inquiry and Research Design: Choosing Among five Approaches*, 3rd Edn Thousand Okas, CA: Sage Publications.

[B6] Creswell J. (2018). *Research Design: Qualitative, Quantitative, and Mixed Methods Approaches*, 5th Edn Thousand Oaks CA: SAGE.

[B7] Dickson T. Gray T. Mann K. (2008). *Australian Outdoor Adventure Activity Benefits Catalogue.* Available at: https://www.academia.edu/8370828/Dickson_T._Gray_T._&_Mann_K_2008._Australian_Outdoor_Adventure_Activity_Benefits_Catalogue_http_outdoorcouncil.asn.au_doc_OutdoorActivityBenefitsCatalogueFinal270808.pdf

[B8] Enoch M. R. Dixon M. R. (2017). The use of a child-based acceptance and commitment therapy curriculum to increase attention. *Child Fam. Behav. Ther.* 39 200–224. 10.1080/07317107.2017.1338454

[B9] Evans G. W. (2006). Child development and the physical environment. *Annu. Rev. Psychol.* 57 423–451. 10.1146/annurev.psych.57.102904.190057 16318602

[B10] Fletcher L. Hayes S. C. (2005). Relational frame theory, acceptance and commitment therapy, and a functional analytic definition of mindfulness. *J. Ration. Emot. Cogn. Behav. Ther.* 23 315–336. 10.1007/s10942-005-0017-7

[B11] Gass M. A. Gillis H. L. Russell K. C. (2012). *Adventure Therapy: Theory, Research, and Practice.* New York, NY: Routledge.

[B12] Ghomian S. Shairi M. R. (2014). The effectiveness of acceptance and commitment therapy for children with chronic pain (CHACT) on the function of 7 to 12 year-old children. *Int. J. Pediatr.* 2 195–203.

[B13] Goodman T. A. Greenland S. K. (2009). “Mindfulness with children: working with difficult emotions,” in *Clinical Handbook of Mindfulness*, ed. Didonna F. (New York, NY: Springer), 417–429.

[B14] Greco L. A. Baer R. A. Smith G. T. (2011). Assessing mindfulness in children and adolescents: development and validation of the child and adolescent mindfulness measure (CAMM). *Psychol. Assess.* 23 606–614. 10.1037/a0022819 21480722

[B15] Greco L. A. Lambert W. Baer R. A. (2008). Psychological inflexibility in childhood and adolescence: development and evaluation of the avoidance and fusion questionnaire for youth. *Psychol. Assess.* 20 93–102. 10.1037/1040-3590.20.2.93 18557686

[B16] Hayes S. C. Strosahl K. D. (2004). *A Practical Guide to Acceptance and Commitment Therapy.* New York, NY: Springer 10.1007/978-0-387-23369-7

[B17] Hayes S. C. Strosahl K. D. Wilson K. G. (1999). *Acceptance and commitment therapy: An experiential approach to behavior change.* New York, NY: Guilford Press.

[B18] Jacobson N. S. Truax P. (1991). Clinical significance: a statistical approach to defining meaningful change in psychotherapy research. *J. Consult. Clin. Psychol.* 59 12–19. 10.1037/0022-006X.59.1.12 2002127

[B19] Kessler R. C. Andrews G. Colpe L. J. Hiripi E. Mroczek D. K. Normand S. L. T. (2002). Short screening scales to monitor population prevalences and trends in non-specific psychological distress. *Psychol. Med.* 32 959–976. 10.1017/S0033291702006074 12214795

[B20] Lawrence D. Johnson S. Hafekost J. Boterhoven De Haan K. Sawyer M. Ainley J. (2015). *The Mental Health of Children and Adolescents. Report on the second Australian Child and Adolescent Survey of Mental Health and Wellbeing.* Canberra, ACT: Department of Health.

[B21] Li W. H. C. Chung J. O. Ho E. K. (2013). Effectiveness of an adventure-based training programme in promoting the psychological well-being of primary schoolchildren. *J. Health Psychol.* 18 1478–1492. 10.1177/1359105312465102 23221616

[B22] Lyneham H. J. Sburlati E. S. Abbott M. J. Rapee R. M. Hudson J. L. Tolin D. F. (2013). Psychometric properties of the child anxiety life interference scale (CALIS). *J. Anxiety Disord.* 27 711–719. 10.1016/j.janxdis.2013.09.008 24135256

[B23] Marsh H. W. (1990). *Self-Description Questionnaire.* Campbelltown, NSW: University of Western Sydney Publication Unit.

[B24] Murrell A. R. Steinberg D. S. Connally M. L. Hulsey T. Hogan T. E. (2015). Acting out to ACTing on: a preliminary investigation in youth with ADHD and co-morbid disorders. *J. Child Fam. Stud.* 24 2174–2181. 10.1007/s10826-014-0020-7

[B25] Reinherz H. Z. Paradis A. D. Giaconia R. M. Stashwick C. K. Fitzmaurice G. (2003). Childhood and adolescent predictors of major depression in the transition to adulthood. *Am. J. Psychiatr.* 160 2141–2147. 10.1176/appi.ajp.160.12.2141 14638584

[B26] Rickwood D. J. Deane F. P. Wilson C. J. (2007). When and how do young people seek professional help for mental health problems? *Med. J. Aust.* 187 S35–S39. 17908023 10.5694/j.1326-5377.2007.tb01334.x

[B27] Roza S. J. Hofstra M. B. van der Ende J. Verhulst F. C. (2003). Stable predictions of mood and anxiety disorders based on behavioral and emotional problems in childhood: a 14-year follow-up during childhood, adolescence, and young adulthood. *Am. J. Psychiatr.* 160 2116–2121. 10.1176/appi.ajp.160.12.2116 14638580

[B28] Ruiz F. (2010). A Review of Acceptance and Commitment Therapy (ACT) empirical evidence: correlational, experimental psychopathology, component and outcome studies. *Int. J. Psychol. Psychol. Ther.* 1 125–162.

[B29] Scrutton R. A. (2015). Outdoor adventure education for children in Scotland: quantifying the benefits. *J. Adv. Edu. Outd. Learn.* 15 123–137. 10.1080/14729679.2013.867813

[B30] Simon E. Verboon P. (2016). Psychological inflexibility and child anxiety. *J. Child Fam. Stud.* 25 3565–3573. 10.1007/s10826-016-0522-6 27891046 PMC5104759

[B31] Truong S. Ward K. Tracey D. Gray T. (2018). *ACT in the Outdoors: A Program Based on Acceptance and Commitment Therapy and Adventure Therapy.* Sydney, NSW: Western Sydney University 10.4225/35/5a961c414081

[B32] World Health Organization [WHO] (2005). *Atlas: Child and Adolescent Mental Health Resources: Global Concerns, Implications for the Future.* Geneva: World Health Organization.

[B33] World Health Organization [WHO] (2013). *Mental Health Action Plan 2013-2020.* Geneva: World Health Organization.

[B34] Zahra D. Hedge C. Pesola F. Burr S. (2016). Accounting for test reliability in student progression: the reliable change index. *Med. Educ.* 50 738–745. 10.1111/medu.13059 27295478

